# Spatial Proteomics for Further Exploration of Missing Proteins: A Case Study of the Ovary

**DOI:** 10.1021/acs.jproteome.2c00392

**Published:** 2022-09-15

**Authors:** Loren Méar, Thanadol Sutantiwanichkul, Josephine Östman, Pauliina Damdimopoulou, Cecilia Lindskog

**Affiliations:** † Department of Immunology, Genetics and Pathology, Uppsala University, 75185Uppsala, Sweden; ‡ Division of Obstetrics and Gynecology, Department of Clinical Science, Intervention and Technology, Karolinska Institutet and Karolinska University Hospital, 14186Stockholm, Sweden

**Keywords:** missing proteins, antibody-based proteomics, immunohistochemistry, transcriptomics, human proteome, TMA, Human Protein Atlas, ovary, tissues

## Abstract

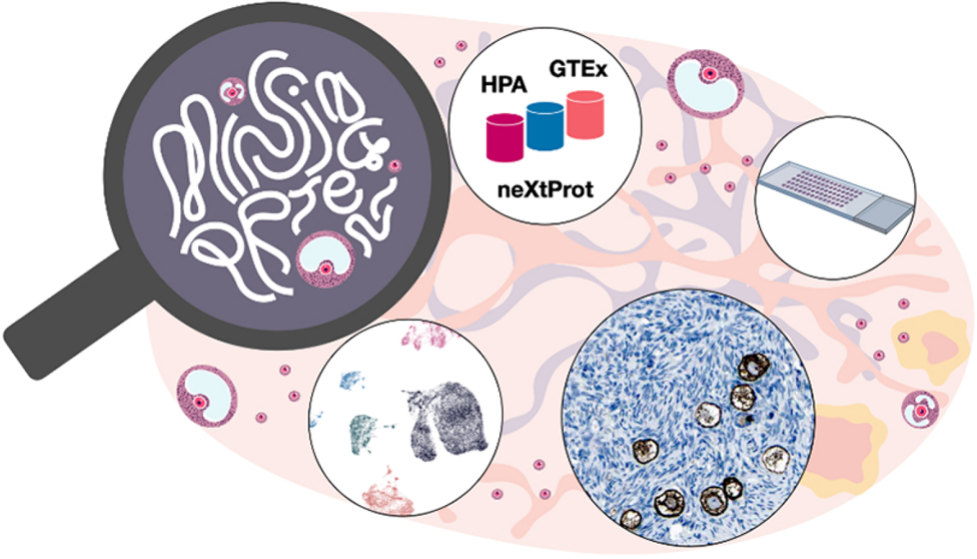

In the quest for “missing proteins” (MPs), the proteins encoded by the human genome still lacking evidence of existence at the protein level, novel approaches are needed to detect this challenging group of proteins. The current count stands at 1,343 MPs, and it is likely that many of these proteins are expressed at low levels, in rare cell or tissue types, or the cells in which they are expressed may only represent a small minority of the tissue. Here, we used an integrated omics approach to identify and explore MPs in human ovaries. By taking advantage of publicly available transcriptomics and antibody-based proteomics data in the Human Protein Atlas (HPA), we selected 18 candidates for further immunohistochemical analysis using an exclusive collection of ovarian tissues from women and patients of reproductive age. The results were compared with data from single-cell mRNA sequencing, and seven proteins (CTXN1, MRO, RERGL, TTLL3, TRIM61, TRIM73, and ZNF793) could be validated at the single-cell type level with both methods. We present for the first time the cell type-specific spatial localization of 18 MPs in human ovarian follicles, thereby showcasing the utility of the HPA database as an important resource for identification of MPs suitable for exploration in specialized tissue samples. The results constitute a starting point for further quantitative and qualitative analysis of the human ovaries, and the novel data for the seven proteins that were validated with both methods should be considered as evidence of existence of these proteins in human ovary.

## Introduction

The Human Proteome Project (HPP) is an international project organized by the Human Proteome Organization (HUPO), with the main purpose to characterize and understand the function of the human proteome.^1^ In this context, the neXtProt knowledge base (www.nextprot.org) annually updates its ranking of human proteins according to evidence of their existence, called Protein Existence (PE).^2^ According to the most recent neXtProt release (2022–02–25), 93.2% of all proteins predicted by the human genome have been experimentally validated (primarily using high-quality mass spectrometry (MS) approaches), and are called PE1. There are still however 1,343 proteins lacking evidence at the protein level and considered missing proteins (MPs). MPs have either evidence of existence at transcript level (PE2), or have been inferred by homology (PE3) or are predicted (PE4). The reason for these proteins still lacking evidence may be due to a low expression level, expression in rare cell or tissue types,^3^ or the cells in which they are expressed only represent a small minority of the tissue. Studying and identifying these MPs constitutes a major challenge due to the need to pinpoint the cells or tissues where they are expressed.

The Human Protein Atlas (HPA) project aims to map all human proteins in cells, tissues, and organs by integrating different omics technologies, including antibody-based proteomics and transcriptomics.^4^ All data generated by the HPA is publicly available on the Web site https://www.proteinatlas.org/, and the database provides a valuable source of information related to human protein expression that can be used to explore MP expression profiles across different tissues at both mRNA and protein levels. Since the MPs may have a low expression level, we decided to focus on a single organ: the ovary as a case study and proof of concept.

The ovary is an extremely dynamic organ and has a crucial role in both endocrine and reproductive systems in women. The main function is to produce hormones and mature gametes, and monthly it undergoes structural changes to release oocytes during reproductive years, from puberty to menopause. The oocyte surrounded by granulosa cells form a specific and functional structure, the follicle. The reserve of prenatally formed nongrowing follicles resides in the surface layer of the ovary, the cortex. As the follicles grow in size, they migrate to the inner part, the medulla, that is composed of blood vessels, loose connective tissue, and nerves. The nongrowing follicles continuously enter the growing pool but only after puberty the folliculogenesis can be completed under the influence of pituitary gonadotropins. The follicles however only constitute a very small proportion of the total number of cells, with the remaining cells making up the ovarian stroma.^5^ The follicle number dramatically declines from birth, when there are 1–2 million primordial follicles, to puberty when only 300,000–500,000 remain. At last, only 500 of them will proceed through the whole folliculogenesis, a process that takes approximately half a year in humans, while the rest will be eliminated by atresia. A recent study on the single cell transcriptome of ovarian cortex in adult women has only been able to recover a few oocytes, approximately 0.2% of the sequenced cells.^5^ Due to the limited number of follicles, their heterogeneous distribution across the ovarian cortex, the dynamic changes in cell composition during the menstrual cycle and difficulties to obtain ovarian samples from healthy women of reproductive age, the transcriptome and proteome of the immatures oocytes during folliculogenesis remain poorly understood. In the standard HPA workflow, ovary is included as one of the tissue types that has been profiled using both bulk mRNA sequencing (RNA-seq) and immunohistochemistry, but most samples are from postmenopausal women, and the presence of follicles in the analyzed samples is extremely rare. Here, we present an integrated approach utilizing the publicly available HPA data to identify suitable MP candidates for further analysis. On the basis of an exclusive collection of tissue samples from women and patients of reproductive age, we profiled the selected proteins in follicles using a stringent immunohistochemistry workflow and compared the results with data from single-cell mRNA sequencing (scRNA-seq). We present for the first time the cell type-specific spatial localization of 18 MPs in human follicles, mainly immature ones within the ovarian cortex, thereby showcasing the utility of the HPA database as an important resource for identification of MPs suitable for exploration in extended tissue samples. The results constitute a starting point for further quantitative and qualitative analysis of the human ovaries.

## Experimental Procedure

### Human Tissue Samples

Anonymized human ovarian tissue samples from three individuals (ages 21, 22, and 30) were collected from gender reassignment patients at Karolinska University Hospital. In agreement with the Declaration of Helsinki, the patients received oral and written information, and signed an informed consent form. Tissues were retrieved from the operation theater and transported to the research laboratory in PBS within 15 min. The cortex was trimmed from medulla, and tissue from both compartments was fixed in formalin and stored in paraffin blocks. The project was approved by the Swedish ethical review authority #2015/798-31/2 and #2021-04563. For further validation of the 18 top candidates, a unique tissue microarray (TMA) of ovarian tissue samples was designed. Human ovarian tissue samples along with tissues from 20 other human organs for antibody optimization were obtained from the Clinical Pathology department, Uppsala University Hospital (Sweden) and collected within the Uppsala Biobank organization. All samples were anonymized for personal identity by following the approval and advisory report from the Uppsala Ethical Review Board (ref nos. 2002-577, 2005-388, 2007-159). Informed consent was obtained from all subjects in the study. The ovarian TMA consisted of 16 tissues from different age groups: nine from reproductive age (age 35–47) and seven in postmenopausal age (age 55–86).

### Protein Profiling

The TMA building, immunohistochemical staining, and digitization of the stained TMA slides have been performed as previously described.^6^ Briefly, TMA paraffin embedded formalin-fixed blocks were cut with waterfall microtomes (Microm H355S, ThermoFisher Scientific, Freemont, CA) at 4 μm and placed on Superfrost Plus slides (Thermo Fisher Scientific, Freemont, CA) to dry at room temperature. Then slides were baked at 50 °C for 12–14 h. Automated immunohistochemistry was performed by using Lab Vision Autostainer 480S Module (Thermo Fisher Scientific, Freemont,CA), as described in detail previously.^7^ Primary antibodies used and their dilution are listed in Table S1. Western blot results based on a standardized set of lysates are publicly available in the HPA database for each of the antibodies used in the study, with the url structure https://www.proteinatlas.org/ENSG00000157999-ANKRD61/antibody using Ensembl ID and gene name as unique identifies. The slides were scanned using the Aperio AT2 (LeicaAperio, Vista, CA) using the 40× objective. All tissue samples were manually annotated by CL for two cell types: oocytes and granulosa cells. Staining intensity has been used as the main annotation parameter using a scale from 0 to 3, based on the standardized HPA workflow:^4^ 0 = not detected; 1 = low; 2 = medium, and 3 = high.

### scRNA Reanalysis

In order to confirm expression of candidates at the single cell level, reanalysis of scRNA sequencing has been performed. The data of human ovarian cortex scRNA derived from four samples (three from cesarean and one from gender reassignment patients) were obtained from ArrayExpress archive (E-MTAb-8381).^5^ A second set of data was downloaded from gene expression omnibus (GSE118127) derived from five ovaries from cancer patients.^8^ First, these data were analyzed separately including filtering, normalization, and clustering using the Seurat V4 package^9^ in R (CRAN). Briefly, cells were removed when they had more than 20% reads mapped to the mitochondrial expression genome and expressed fewer than 500 genes. In our analysis, standard Seurat settings were applied to normalize gene expression data, and the 5,000 most highly variable genes were used to cluster cells. All scatter plots were generated using the UMAP method. Each cluster was assigned an identity based on well-known markers and given a name based on the main cell type in that cluster, including oocytes (such as ZP3 or FIGLA), granulosa cells (AMH), immune cells (CD69 and CD14), endothelial cells (VWF and CD34), smooth muscle cells (MYH11), and stromal cells (DCN). Second, integration of these data sets was performed using the reciprocal PCA tool implemented in Seurat to identify anchors and metadata from the previous analysis used to identify the cell types. To visualize the expression level across the different ovarian cell types, Seurat’s DotPlot function was used.

## Results

### Target Genes and Candidate Selection

**Figure 1:**
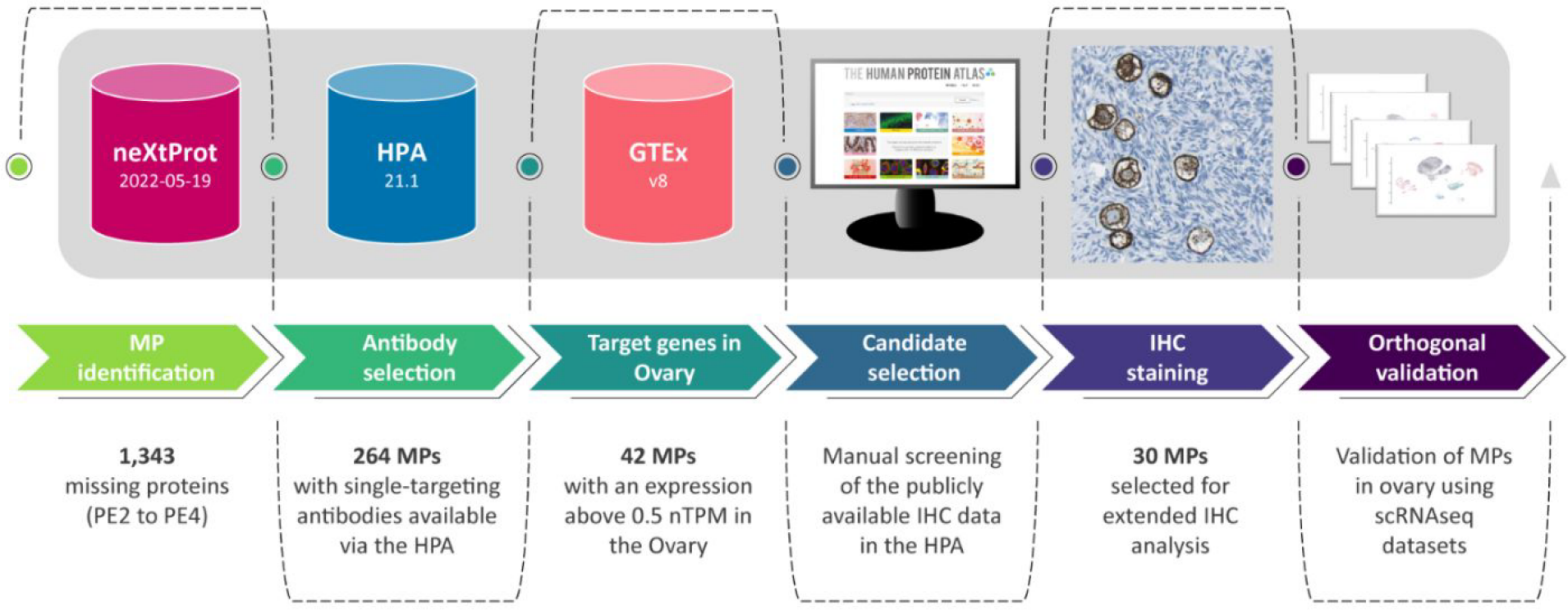
Workflow illustrating the strategy implemented to select the candidates.

We utilized the HPA database to identify and explore suitable MPs for extended analysis in human ovaries (Figure 1).

**Figure 2:**
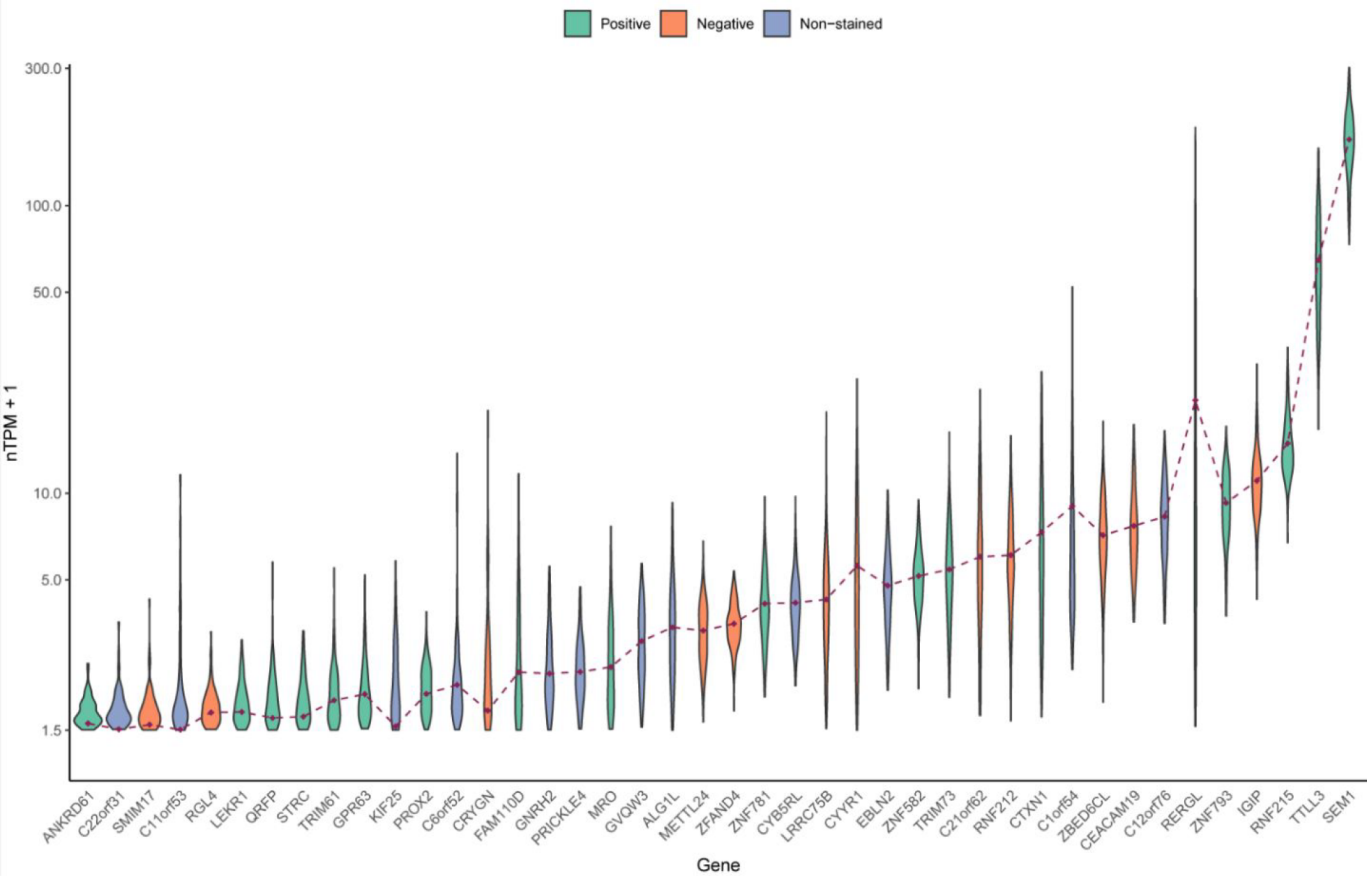
Violin plots displaying the expression level (nTPM+1) of the 42 candidates among the ovarian GTEx samples. The brown dots represent the consensus value from GTEx and were used as threshold values. The ones excluded for the IHC analysis (*n* = 12) are in blue, those with a negative or indistinct staining in the initial test staining (*n* = 12) are in orange, and those with a distinct positive staining in the ovary (*n* = 18) in green.

In accordance with the latest release of neXtProt (2022–02–25), 1,343 proteins are considered as MPs (PE2 to PE4). Version 21 of the HPA contains immunohistochemistry-based protein data for 15,323 protein-coding genes that have been ranked based on antibody reliability using specific criteria adapted from the International Working Group for Antibody Validation (IWGAV),^10^ as described previously.^11^ In the present investigation, we however decided to neglect the present ranking of the antibody reliability during the first filtering, since it seems likely that the ranking was based on conclusions without considering the expression pattern in oocytes. This may mean that antibodies with lower reliability scores would indeed be considered reliable and generate the correct staining pattern if samples from women of reproductive age with the presence of follicles are used. In addition to the publicly available immunohistochemistry data, the HPA has access to >25,000 antibodies for which tissue stainings have not been published, and some of these may previously have been considered unreliable due to the lack of correct samples for optimization. Here, we used the list of 1,343 MPs to filter proteins with at least one available antibody either publicly available via the HPA, or unpublished in the HPA internal system. Only single-targeting antibodies were considered, i.e., expected to target a single protein based on having low sequence identity (maximum 60%, with the vast majority having < 40%) to all human transcripts, except for those corresponding to the gene of interest. There were in total 264 MPs with single-targeting antibodies available via the HPA. Next, we filtered the identified targets based on bulk mRNA expression in ovary, according to a consensus classification combining internally generated RNaseq data from the HPA consortium^4^ with RNaseq data from the Genotype-Tissue Expression project (GTEx).^12^ The RNaseq data for ovary altogether comprised samples from 183 women, out of which a majority (*n* = 107) were from women of postmenopausal age (>50 years) and few of them (*n* = 36) were women younger than 40 years old. Since the total number of follicles contributing to the mRNA pool in the consensus ovary data set are expected to be very low, we decided on a low detection cutoff (nTPM > 0.5). The total number of MPs with available single-targeting antibodies and an mRNA expression in the ovary above 0.5 nTPM was 42, all belonging to the PE2 category of MPs, with previous evidence at transcript level (Figure 2; Table S1). Detailed information regarding the MPs (e.g., chromosome location and PE status) is also provided in Table S1. As seen in the violin plot (Figure 2), expression levels for several candidates were low, and increasing the detection cutoff to 1.0 nTPM would result in 30 candidates, thus the lower cutoff of 0.5 nTPM was chosen.

It should be noted that SEM1 has two unique identifiers in UniProt/neXtProt, out of which one is PE1 (SEM1 26S proteasome complex subunit) and one is PE2 (Putative protein SEM1, isoform 2). The two proteins however have the same Ensembl ID (ENSG00000127922), and since the HPA database is built upon the human protein-coding genes according to Ensembl, it is not possible to determine which of the two proteins is detected by the antibody. We still decided to include SEM1 in the present investigation. Of the 42 MPs, 12 candidates were excluded based on screening of the publicly available immunohistochemistry data in the HPA, either showing an evenly distributed staining pattern across other tested human tissues and cells or a lack of positive staining in follicles present in the samples. The remaining 30 proteins were selected for an extended immunohistochemistry analysis.

### Antibody-Based Protein Profiling of MPs in Human Ovary

**Figure 3:**
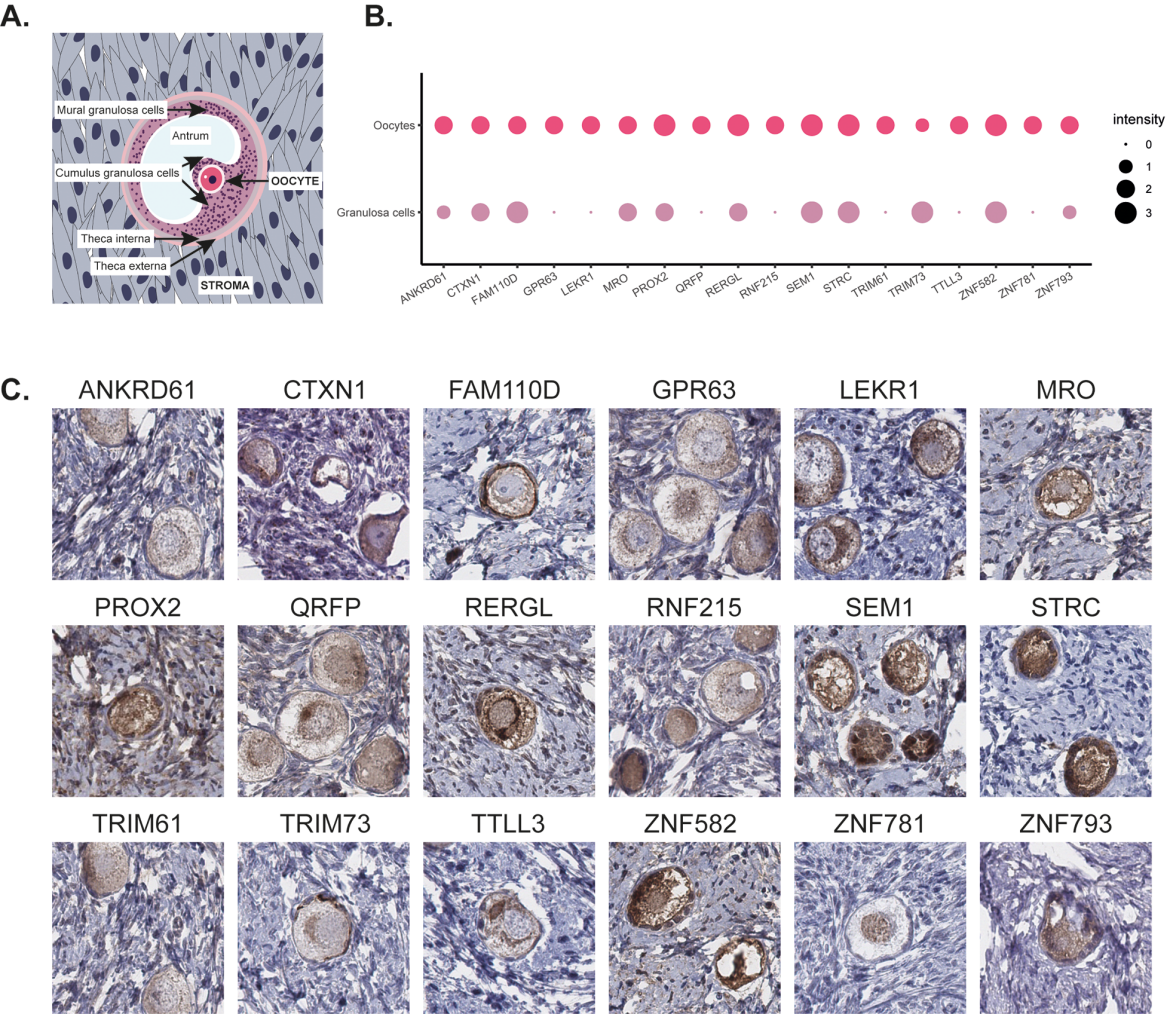
A schematic overview of ovarian tissue (A). Dotplot summarizing the immunohistochemical staining pattern in oocytes and granulosa cells with the size of dots representing the intensity of the staining (B). Representative images of immunohistochemical stainings of human ovary targeting 18 different MPs (C). The brown color indicates antibody binding. Eleven proteins (ANKRD61, GPR63, LEKR1, PROX2, QRFP, RERGL, RNF215, TRIM61, TTLL3, ZNF781, and ZNF793) showed most prominent protein expression in oocytes, two proteins (FAM110D and TRIM73) showed highest expression in granulosa cells, and five proteins (CTXN1, MRO, SEM1, STRC, and ZNF582) showed equally strong expression in both oocytes and granulosa cells. FAM110D showed cytoplasmic and membranous staining, ZNF781 nuclear staining, SEM1 and RERGL displayed a combination of both cytoplasmic and nuclear staining, and the remaining proteins were exclusively expressed in the cytoplasm.

The 30 MPs selected for extended immunohistochemistry analysis were first stained on a large section of human ovarian cortex, corresponding to one individual of reproductive age (age 21), together with a TMA comprising 20 other normal tissues and organs for determination of antibody specificity. In this initial staining, 12 MPs were excluded either due to absence of staining in follicles, diffuse or indistinct staining, or evenly distributed staining across other tissues and cells. The remaining 18 MPs showed distinct staining in follicles and were further evaluated on ovarian samples from a total of 19 individuals, out of which 12 were from women in reproductive age. Figure 3A shows a schematic overview of the histology of ovarian tissue. A summary of the staining patterns in oocytes and granulosa cells is presented in Figure 3B, and representative images of immunohistochemical staining patterns are shown in Figure 3C. The staining was consistent between the individuals both in terms of cell type specificity and staining intensity, and in cases where additional positivity was observed in structures outside follicles, such as stromal cells or endothelial cells, no difference was noted between the women of reproductive age and the women of postmenopausal age for any of the 18 candidates. Eleven of the proteins (ANKRD61, GPR63, LEKR1, PROX2, QRFP, RERGL, RNF215, TRIM61, TTLL3, ZNF781, and ZNF793) showed most prominent protein expression in oocytes, in some cases accompanied by weaker staining in granulosa cells, while two proteins (FAM110D and TRIM73) showed highest expression in granulosa cells. Five proteins (CTXN1, MRO, SEM1, STRC, and ZNF582) showed equally strong expression in both oocytes and granulosa cells.

The 18 MPs analyzed here were previously largely uncharacterized. Only two proteins had previous data on functions related to ovary or reproduction in humans. Gene variants of LEKR1 have been associated with ovarian cancer risk^13^ and low birth weight,^14^ while MRO is transcribed in males before and after differentiation of testis. MRO transcripts have also been detected in the postmenopausal ovary,^15^ but no study has previously described the presence in follicles. Four proteins have been identified to carry out ovary-specific or reproduction-related processes in other species, including the orphan receptor GPR63 that has been described to participate in egg production in ducks,^16^ and the neuropeptide QRFP that in rats is suggested to enhance LH secretion directly at the level of the pituitary (Navarro2006). Other examples are the hydrolase RERGL, identified as one of the genes in fish eggs that were affected by radiation after the Chernobyl Nuclear Power Plant accident^17^ and the ligase TTLL3 which is essential for blastocyst formation in cows.^18^ None of these proteins has previously been mentioned in the context of human ovary or reproduction. The remaining proteins were either suggested to be involved in transcription regulation (PROX2, ZNF582, ZNF781, and ZNF793) or metal binding (RNF215, TRIM61, and TRIM73), had limited data on diverse functions not directly related to reproduction (CTXN1, SEM1, and STRC), or were completely uncharacterized (ANKRD61 and FAM110D). In the present investigation, we could show that all these 18 MPs showed protein expression in human ovarian follicles.

### Validation of MPs in Ovary Using scRNAseq

**Figure 4:**
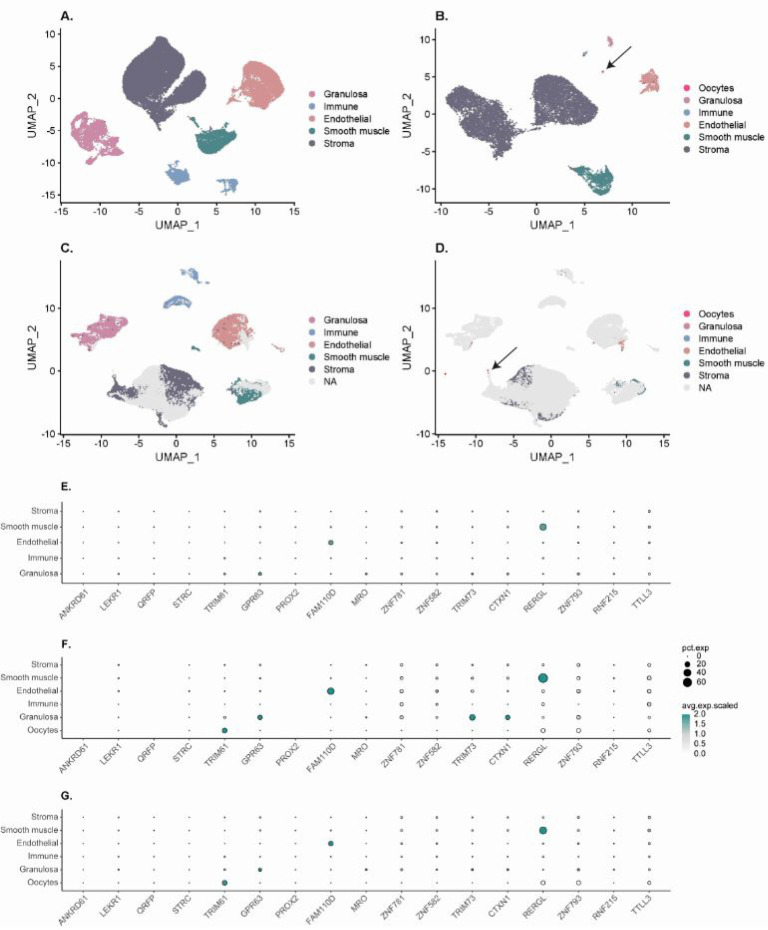
Overview of the scRNAseq analysis. UMAP plots of the GSE118127 data set (A), the E-MTAb-8381 data set (B), and the integration of the two data sets with colored cells from the GSE118127 data set (C) or the E-MTAb-8381 data set (D) in order to highlight the contribution of each data set in the integrated one. Dotplots displaying the scaled expression levels of the 18 top candidates from the GSE118127 data set (E), the E-MTAb-83831 data set (F), and the integrated one (G). The size of the dots represent the percentage of cells expressing the marker in each cluster (pct.exp), and the color scale represents the average expression level. ANKRD61, PROX2, and QRFP expression values were not available in the E-MTAb-83831 data set (F), and SEM1 expression values were not available in any of the two data sets and are not displayed at all in the dotplots.

In order to validate the single cell expression obtained by spatial proteomics, we compared the results with single cell transcriptomics data based on scRNAseq. Two different data sets were reanalyzed, first independently and then by integration. In the first data set GSE118127,^8^ 29 distinct clusters with a total of 42,509 cells were available for single-cell profiling. The clusters were regrouped to obtain five main ovarian cell populations: granulosa cells (5,449 cells; 12.8%), immune cells (2,688 cells; 6.3%), endothelial cells (7,915 cells; 18.6%), smooth muscle cells (3,877 cells; 9.1%), and stromal cells (22,664 cells; 53.2%). No oocytes were retrieved in this data set (Figure 4A). In the second data set, E-MTAb-83831,^5^ 12,157 cells were analyzed, corresponding to 14 clusters that were regrouped into six main cell types: oocytes (17 cells; 0.1%), granulosa cells (138 cells; 1.1%), immune cells (40 cells; 0.3%), endothelial cells (623 cells; 5.1%), smooth muscle cells (1,165 cells; 9.6%), and stromal cells (10,174 cells; 83.7%) (Figure 4B). The integration of the two data sets confirmed the overlap between the main cell ovarian population across the tissues, with a majority of cells corresponding to ovarian stroma (60%) and only a few oocytes (0.03%) (Figure 4C, D). In order to confirm our immunohistochemistry results, the expression of the top 18 genes was evaluated across the different single cell clusters and data sets (Figure 4E–G, Table S2). Among the 18 genes, four had N/A values in either one data set (ANKRD61, QRFP, and PROX2) or both (SEM1), and consequently, we were not able to draw any conclusions for these proteins. Furthermore, some genes displayed very low (STRC) or nonspecific expression (ZNF781 and ZNF582) that prevented us from confirming our previous results. Four candidates were based on the scRNAseq shown to be expressed in oocytes and corroborated the IHC findings (RERGL, TRIM61, TTLL3, and ZNF793). RERGL also showed expression at a high level in smooth muscle clusters, as described in the publication associated with the E-MTAb-83831 data set,^5^ in which it is used as a marker for both smooth muscle and pericytes. In granulosa cell clusters, TRIM73 is expressed as expected, as well as CTXN1, MRO. Two other genes (RNF215 and GPR63) are also expressed although they were specific to oocytes at the protein level. The remaining candidates seemed either to be expressed in endothelial cells (FAM110D) or in both granulosa and stromal cells (LEKR1). Interestingly, FAM110D showed additional high expression in endothelial cells also at the protein level and could thus be partly confirmed by the scRNAeq data, but the staining in granulosa cells which is of importance for the current study could not be validated. The discrepancy between mRNA and protein levels could either be due to mixed single cell clusters and a very limited number of cells expressing the gene at low levels. Another possible explanation is unspecific antibody binding. Nevertheless, the single cell type specific expression could be confirmed at both the mRNA and protein level for seven proteins.

## Discussion

A decade after the initiation of the HPP project, a high-stringency blueprint of the human proteome was released,^19^ whereby ≥1 protein product from each protein-coding gene had been identified for 90.4% of the human proteome using established and reliable standards for mass spectrometry-based protein detection. Two years later, the count now stands at 93.2%, leaving 1,343 proteins that still lack evidence of existence at the protein level. It is anticipated that the remaining MPs require sampling of rare tissues or cells taking into consideration temporal changes in expression, and the limit of detection may be particularly challenging.

While mass spectrometry constitutes the standard method for detecting and quantifying a targeted set of human proteins in a sample, the technology has limitations when it comes to lowly expressed proteins. It is likely that detection of some of the remaining MPs require analysis using other methodologies or further technological advancements to avoid bias toward highly expressed proteins. For ovarian tissue, there is only one previous study based on mass spectrometry,^20^ further emphasizing this tissue as particularly challenging. Here, we used an integrated omics approach to identify and validate MPs in human ovarian tissue with spatial and single-cell resolution. Immunohistochemistry is the standard method for spatial proteomics and has the advantage of sensitive detection in smaller subsets of cells, allowing for analysis of human proteins with a single-cell resolution. We were able to localize 18 MPs to specific structures in human ovarian tissue, thereby constituting a first step toward understanding their function and providing the basis for further analysis using other methodologies.

One challenge that needs to be addressed in the context of immunohistochemistry is the need for stringent validation of antibodies, in order to ensure that the antibodies are binding to the intended targets. In the HPA workflow, methods for antibody validation build upon strategies suggested by the IWGAV consortium,^10^ which for immunohistochemistry approaches means two possible approaches: (1) orthogonal validation, comparing the expression pattern using an antibody-independent method across multiple samples that should express the protein at different levels or (2) independent antibody validation, confirming the expression pattern across multiple samples with the use of another antibody that binds to a nonoverlapping sequence of the same protein. For the antibodies used in the present investigation, the defined criteria for orthogonal validation^11^ could not be met due to low expression levels in the ovary and the small number of follicles present for comparison, both in the bulk transcriptomics and scRNAseq data. Nevertheless, all 18 antibodies analyzed were also stained on a TMA together with 20 other human tissue types to optimize the antibody dilution and confirm that the staining in other tissue types was not contradictory to RNAseq values. While it is not enough for orthogonal validation, it will allow us to upgrade the reliability score to “supported” in the upcoming version of the HPA database. For none of the 18 MPs tested here, there was additional suitable independent antibodies available via the HPA that could be used for independent antibody validation.

Recently, a novel technology called Deep Visual Proteomics (DVP) was introduced, which combines submicron-resolution imaging, single-cell phenotyping based on artificial intelligence (AI), and isolation with an ultrasensitive proteomics workflow.^21^ The technique builds upon laser microdissection and constitutes an important complement to previously established methods such as standard mass spectrometry, imaging mass spectrometry methods with lower pixel resolution, as well as spatial proteomics methods based on antibody-based imaging. This could add another layer in the quest for identification of MPs. To date, this novel method has however not been applied to ovarian tissue.

Proteins are the main molecules carrying out the functions of human cells and also constitute the targets for most pharmaceutical drugs. Generating a complete map of human proteins across tissues and cells is thus crucial for future precision medicine efforts. Another important biomolecule is mRNA and despite the correlation between mRNA and protein may not always be perfect; however, measurements of mRNA provide a starting point for screening of suitable samples that can be further validated with proteomics. Here, we used bulk transcriptomics data in the first filtering of suitable targets, and while the limit of detection may be considered arbitrary in the context of detecting genes expressed in a small subset of cells, it aids in narrowing down the list to candidates more likely to be identified with antibody-based imaging. Dramatic improvements in single cell RNA sequencing (scRNA-seq) have lead to an increased awareness of the importance to provide a detailed characterization of the human building blocks. This has resulted in multiple worldwide single-cell mapping efforts including the Human Cell Atlas (HCA),^22^ the Chan Zuckerberg Initiative (CZI),^23^ the Human BioMolecular Atlas Program (HuBMAP)^24^ (funded by the National Institute of Health (NIH)), the Human Cell Landscape,^25^ as well as a large number of other research studies mapping human tissues in health and disease. Reproductive organs are however still underrepresented in such efforts and constitute a particular challenge due to temporal expression. In the present investigation, we used ovarian tissue scRNAseq data from two different studies to validate the expression patterns observed by immunohistochemistry. We were able to validate that four of 18 MPs in immature oocytes are enclosed in nongrowing cortical follicles (TRIM61, ZNF793, TTLL3, and RERGL) and three in granulosa cells (TRIM73, CTXN1, and MRO). Low expression levels were observed in granulosa cell clusters for RNF215 and GPR63 contradicting our immunohistochemistry results. Contradictory data was also observed for FAM110D and LEKR1, with a discrepancy of highest expressed cell types at the mRNA or protein level. For seven proteins (ANKRD61, PROX2, QRFP, SEM1, STRC, ZNF781, and ZNF582), a comparison between the data sets was not possible due to missing values, weak or unspecific expressions. Considering the very limited number of cells analyzed, especially for oocytes (*n* = 17) and the shallow sequencing level, the results should however be treated with caution and can therefore not be considered orthogonal validation. Nevertheless, the scRNAseq analysis confirmed the scarcity of oocytes in comparison to the stroma, and it is likely that future efforts and technology advancements will lead to novel studies with an improved yield of cells that can be used for integrated studies combining transcriptomics with proteomics. One of the disadvantages with both bulk transcriptomics and scRNA-seq is the loss of transcript location in relation to the tissue geography. Powerful technologies for mRNA detection while preserving intact tissue architecture are spatial transcriptomics,^26^ or in situ hybridization (ISH) using RNAScope.^27^ Studies validating data across disciplines are rare, and there are no effective means on how multimodal data should be integrated to leverage novel insights on specific physiological processes. For a full understanding of the human building blocks and determining the function of each human protein, integration across data sets and platforms is key, taking into consideration the specific characteristics of each method and data set, allowing for validation across disciplines. Future efforts should thus focus on combining such methods using the same samples.

In the present study, we present the single cell-type specific localization of 18 MPs in human ovarian cortical tissue based on spatial proteomics, out of which seven (CTXN1, MRO, RERGL, TTLL3, TRIM61, TRIM73, and ZNF793) could be validated using scRNAseq. The data for these seven proteins in human ovary should be considered when curating their evidence of existence at the protein level. This case study thus suggests that the approach of integrating transcriptomics with antibody-based proteomics constitutes an attractive starting point for identification and further exploration of MPs. The identified candidates should be further validated using targeted approaches of human ovaries, with a particular focus on the cell types present in follicles also considering the dynamic changes that take place during follicle growth.

## Supporting Information Available

List of candidates with available information on the gene, mRNA, and protein level, including chromosome location and antibody dilution used in the analyses XLSX)
Overview of single cell mRNA expression levels in different cell types across three different data sets for the 18 proteins analyzed in the study (XLSX)
